# Radical Cure of Hæmorrhoids

**Published:** 1872-05

**Authors:** A. W. Calhoun

**Affiliations:** Berlin, Prussia


### FOREIGN CORRESPONDENCE.

### RADICAL CURE OF HÆMORRHOIDS.

BY A. W. CALHOUN, M. D.

No exaggeration, perhaps, will be made, if I say that one-half, if not two-thirds of every community, are sufferers, to a greater or less extent, from Piles. It is one of the commonest and most harrassing diseases, to which we are heirs, and although but seldom attaining an even slightly life-threatening degree, yet, with a few exceptions, each individual thus affected, would most readily and willingly accede to any reasonable plan, of whatever severity, that promised permanent relief from this most disturbing and disagreeable and frequently most painful of all bodily ills.

The fact that every man naturally possesses, in himself, one or more of the numberless causes of this affection, is a clear explanation of its conspicuous frequency, for the anatomical construction of the parts in question, show the ease with which they, upon a very slight stimulus, depart from their normal and healthy state.

The remarkable predominance of this disease amongst males over females, is, as is generally known, due to the fact that the tendency to congestion of the lower bowels, is counteracted in females by the regular menstrual flow, while in man, no natûral outlet being afforded to the pent up blood, or rupture of some one or more of the hæmorrhoidal vessels occur, often upon the least exciting cause, and that most unpleasant companion, the PILES, is called into existence, which, if not properly noticed, and with similar causes remaining, increase in size and number and pain, till the individual's life is one of constant torture.

The more wealthy classes are the especial victims of this disease, since, as a rule, with their habitual high living and other luxurious habits, they combine a more or less sedentary life, all of which are well known to be amongst its most pre-disposing causes ; while on the other hand those who walk more humbly in life and are forced, by the nature of their avocations, and perhaps, want of means, to make regularity and activity, of prime consideration in their daily routine, can congratulate themselves upon their comparative exemption from this "hanger on" to their, in some respects, more fortunate brothers. It is interesting to know that amongst the many other hereditary bodily ailments, this disease takes also its stand, as many convincing examples of its transmission from family to family are narrated, and Prof. Langenbeck, of the University of Berlin, gives his opinion to the same effect, and quotes striking incidents which have come under his own eye. The history of a man operated upon, a few years ago, shows that the affection was confined, not only to himself, since early childhood, but that his father and several other members of the family were similarly affected, the disease beginning mostly in each one when quite young.

Of the special causes of hæmorrhoids, or of the innumerable methods of treatment in use for their temporary alleviation or complete dissipation, I have nothing to say, in a short article of this kind, but come immediately to the actual subject under consideration.

During the present winter, a very large number of both internal and external hæmorrhoids have been operated upon in the clinic of Prof. Langenbeck, all in the same manner, viz: by the "Actual Cautery," and all with successful cures. In fact, of whatever size, or shape, or location of the tumors, no other treatment than that of the red-hot iron, has been practiced for a series of years, in this hospital, which fact alone, is an item in its favor, since, in an institution of this kind, that operation would certainly be followed, which has proven, after long experience, of most permanent benefit to patients.

The bowels are to be freely opened by a laxative, on the day previous to the operation, as they are to be firmly "locked up" for several days thereafter. Of course any local or constitutional obstruction to the operation must be previously set aside by appropriate means. Chloroform is administered, and the patient placed in the position as for Lithotomy, and with double hooks or ring forceps, the tumor drawn sufficiently out. If the hæmorrhoids are very large and extending, as they not unfrequently do, almost entirely around the circumference of the rectum, a part at a time, probably one-half, is seized by "Langenbeck's Clamp" at the base of the tumor. This clamp consists of a somewhat scissors-shaped instrument, with each blade about one-half inch in width at their widest part, and slightly hollowed from the outer to the inner edge, so that when the blades are closed their surface presents a moderately excavated appearance. The inner edge of each blade is roughly grooved, and fit one to the other, and prevents the slipping of any portion of the piles when grasped. The end of the handles is also furnished with a fastener, which holds the instrument in position when applied. Wet cloths are spread underneath the blades, and the "Terrum Candeus" applied to that part of the tumor within the hold of the clamp, burning it down to the instrument. The peculiar form of the clamps and the protection with the wet cloths, wards off any injury to the neighboring parts, that might otherwise result from the proximity of the hot iron, or from the heated fluid from the tumor. Each portion is thus seized and treated till the whole is destroyed, when the parts are gently sponged and oiled, and the bowel, which is more or less protruding, carefully returned.

The after-treatment is quite simple and purely anti-phlogistic; small doses of opium are given to keep in check, for several days, the action of the bowels, and to prevent any pain, and in this hospital (the Royal Surgical), daily injections of a small quantity of oat mucilage are used to keep down local inflammation. Where desirable, the same amount of olive oil, about half an ounce, can be injected alternately with the oat mucilage. If severe inflammation should arise, in addition to the above treatment, India-rubber bags of ice should be applied to the anus and the parts around.

This operation sometimes produces great irritation of the bladder and occasionally stricture at the neck, causing retention of urine, but it can always be relieved by the catheter. This occurred in a case I had the opportunity of observing, where it was necessary to draw off the water by means of an instrument, two or three times a day, for several days in succession.

Hæmorrhage, during this operation, is very slight indeed, and of no consequence, and any after-bleeding is so rare that it is never anticipated. Yet, in the case just mentioned above, the patient passed considerable quantity of *arterial* blood for twelve successive days at each stool, although every care was taken to keep the bowels in a loose condition, and thereby prevent any undue pressure or strain upon the rectum during the passage of the feces. Not only the unusual bleeding was of interest here, but also the arterial character of it, since almost invariably, in all hæmorrhoids, the hæmorrhage before or as a consequence of an operation, is venous. Yet, as before said, any bleeding whatever, after this method, is an absolute rarity, and its absence is therefore claimed as one of the many advantages of the operation.

Cures, by means of the ligature, ecraseur, fluid caustics, etc., etc., except where the disease shows itself in a very slight and unimportant degree, are so seldom resorted to, that they may be considered in a manner discarded, for amongst their good points is interspersed much that is evil. On the other hand, the "Ferrum Candeus" is suitable to almost every case, of whatever size, character or position, and combines in itself most of the good of all the other modes.

Among the large number of applicants to this clinic during the last four or five months, for riddance from this trouble, have been all varieties and stages of the disease, and without an exception, the within described plan has been carried out with as near as possible, perfect or radical cures, the patients, as a rule, being kept under view for four or five weeks after the operation.

The simplicity and easy performance of this operation, with its satisfactory results, have induced me to write this short article, with the hope that it may suggest to those having such affections coming before them, an idea by which good may result, both to patient and physician. And if it so succeeds, I shall be fully remunerated for the few hours spent in its preparation.

BERLIN, PRUSSIA, March 29th, 1872.

